# Urachal Mucinous Cystic Tumour of Low Malignant Potential: A Case Report With Literature Review

**DOI:** 10.7759/cureus.90905

**Published:** 2025-08-24

**Authors:** Abdelrahman Bashar, Mohamed Aabdeen, Jalaja George, Robyn McCrone, Mohammed El-Hassan

**Affiliations:** 1 Urology, Calderdale and Huddersfield NHS Foundation Trust, Huddersfield, GBR; 2 Histopathology, Calderdale and Huddersfield NHS Foundation Trust, Huddersfield, GBR

**Keywords:** cdx2/ck20+, incidental bladder mass, mctlmp, mucuria, rare bladder neoplasm, surgical excision, urachal mucinous cystic tumor

## Abstract

Urachal mucinous cystic tumours of low malignant potential (MCTLPs) are exceedingly rare epithelial tumours. MCTLPs arise from the remnants of the urachus and are characterised by the presence of intestinal-type mucinous epithelium with low-grade cytologic atypia without stromal invasion, which differentiates them from mucinous adenocarcinomas. These tumours are often asymptomatic and found incidentally on imaging done for other reasons; symptoms, however, may include mild abdominal pain/discomfort, storage lower urinary tract symptoms (LUTS) or mucusuria. The treatment is surgical excision, which carries favourable cure rates. We hereby present a case of a 38-year-old man who had an incidental finding of an intravesical cystic lesion on ultrasound done as part of the work-up for newly diagnosed hypertension. The only symptom the patient described in hindsight was an occasional discharge of a gelatinous material in his urine. His work-up also included a diagnostic flexible cystoscopy and a computerised tomography (CT) scan with a delayed urographic phase, which confirmed the finding of a urachal cyst. Histopathological examination following surgical excision confirmed the presence of a rare low-grade urachal MCTLP.

## Introduction

During early foetal development (first trimester), the urachus, derived from the allantois, functions as a conduit for the elimination of waste products from the foetal bladder [1]. This process typically ceases by the second trimester as the urachus undergoes obliteration [2]. However, incomplete closure of the urachal lumen to obliterate appropriately can result in a spectrum of anomalies including urachal sinus, urachal diverticulum, patent urachus, and urachal cyst [3]. Surgical excision is usually warranted for persistent urachal remnants due to the potential risk of complications, such as infection, fistula formation, haemorrhage, obstruction of adjacent viscera, and even malignant transformation [4].

Urachal cancers have a five-year survival rate of 45%, but this is considered to largely be due to their discrete nature which can often cause delays in diagnosis, and many are identified at advanced stages [5]. The most common types of urachal tumours are glandular with adenocarcinomas being the most prevalent type and mucinous cystic tumours (MCTs) are the most common subgroup [6]. Urachal mucinous tumours account for only 0.17% of all bladder cancers [7]. It has been reported that only 26 cases of low-grade MCTs of the urachus have been documented in the literature [8]. Certain cases suggest the potential for dysplastic changes, which may lead to severe complications such as pseudomyxoma peritonei [9]. Reassuringly, there is no evidence in the literature to indicate recurrence of low-grade MCT following surgical excision, supporting this approach as an effective and definitive treatment strategy [10]. The current literature demonstrates successful excision of urachal remnants via open, laparoscopic and robotic approaches. There is no substantial literature to support any of these having significantly better outcomes, therefore, it is largely up to the discretion of the operating surgeon [11].

## Case presentation

An otherwise fit and healthy 38-year-old gentleman was initially referred to our Urology service with an incidental finding of a cystic lesion around the anterior wall of his bladder detected on an ultrasound scan done as part of the work-up for newly diagnosed hypertension. He remained asymptomatic apart from seeing occasional gelatinous material discharge in his urine. By way of background, he doesn’t seem to have any other known medical comorbidities apart from his recently diagnosed hypertension. 

**Figure 1:**
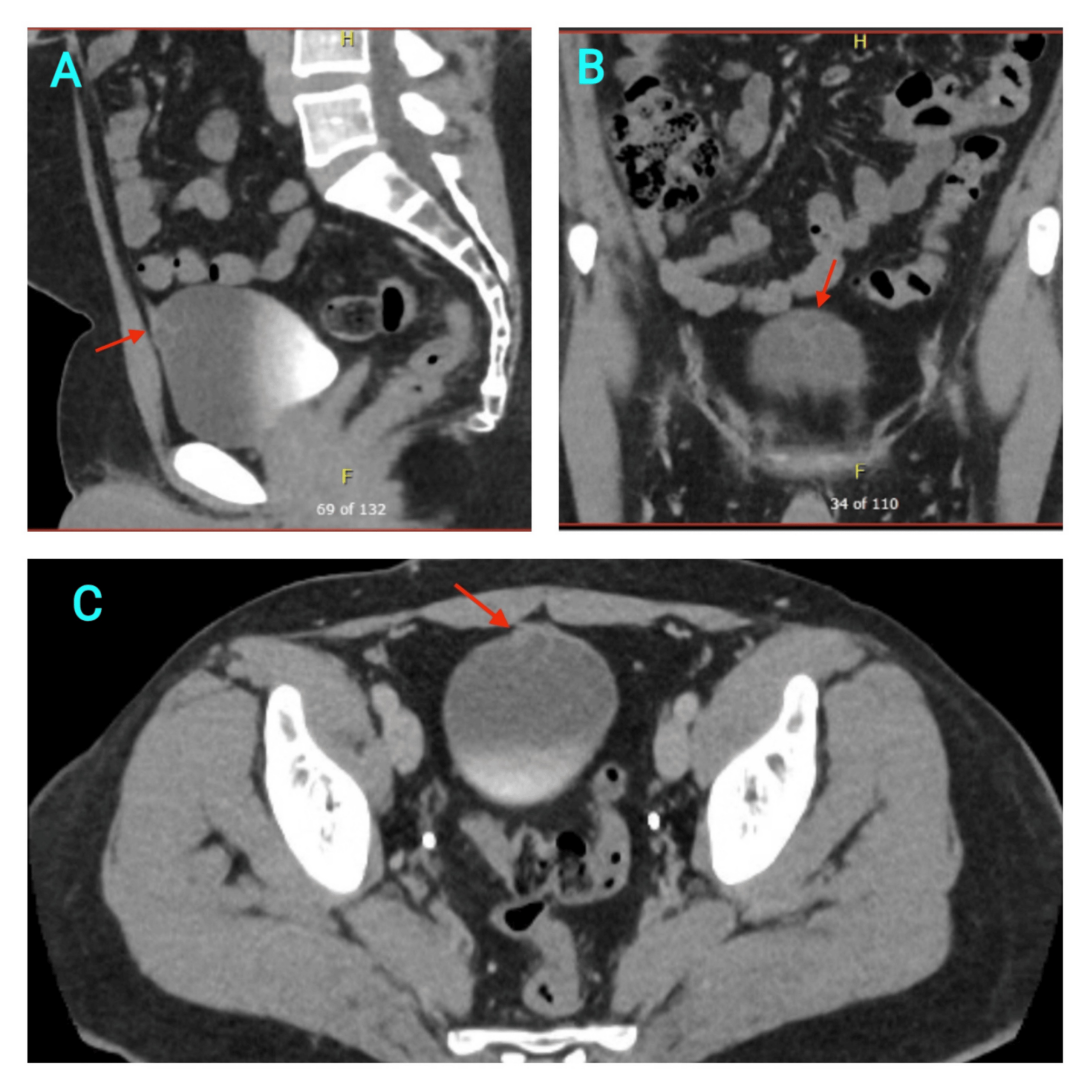
Contrast-enhanced CT scan of the abdomen and pelvis with a delayed urographic phase showing the urachal cyst (red arrows) in three different planes including sagittal (A), coronal (B) and axial (C) planes.

He was reviewed in the Urology One-Stop clinic and had a diagnostic flexible cystoscopy, which confirmed a 3 cm cystic-looking mass at the anterior wall of the bladder towards the dome. A subsequent computerised tomography (CT) scan with a delayed urographic phase confirmed a small bi-lobed cystic lesion in the dome of the urinary bladder at the attachment of the urachus measuring 15x20mm, suggesting an intravesical urachal cyst with no solid components (Figures 1A-1C).

The case was then discussed at the urology multidisciplinary team (MDT) meeting and considering the potential risk of malignant transformation, the most resolute option was felt to be excision of the cystic structure. The patient underwent successful open excision of the urachal cyst following a diagnostic rigid cystoscopy and urethral catheter insertion. There were no immediate complications, and the patient was discharged on the first postoperative day in very good condition with the catheter in situ, which had been removed on the 10th postoperative day. The histopathological examination of the excised urachal cyst revealed a urachal MCTLP. This was subsequently rediscussed in the Urology MDT meeting and the agreed MDT plan was to offer the patient a follow-up plan consisting of a surveillance CT scan with a delayed urographic phase and a check flexible cystoscopy in 12 months to check for any recurrence.

Histological findings

Grossly, the specimen consisted of a nodular piece of fatty tissue measuring 40 x 18 mm. On sectioning, a 20 mm biloculated cyst containing mucinous material was identified, with no solid areas observed. The entire specimen was processed for microscopic examination.

**Figure 2:**
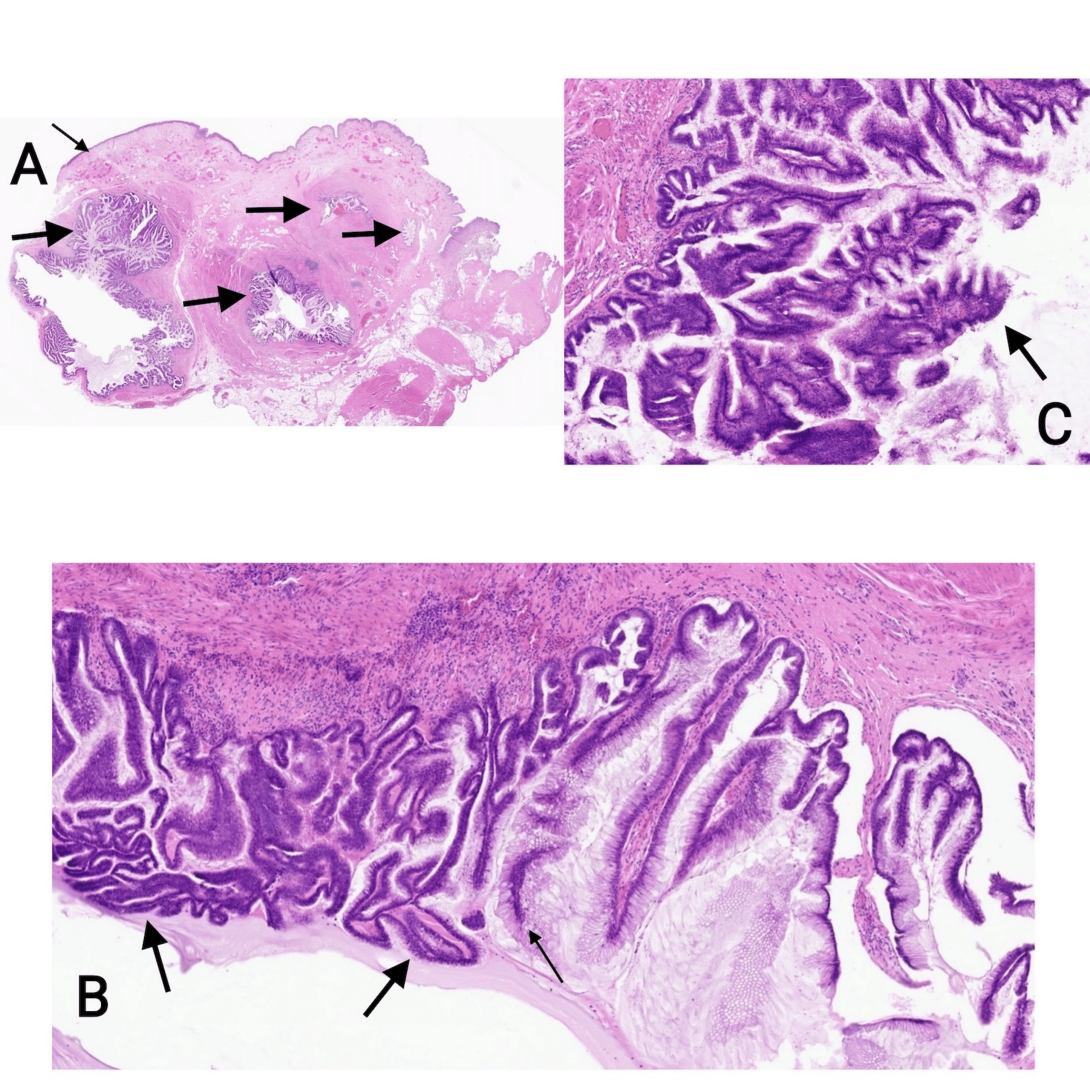
Haematoxylin and eosin-stained sections A. HE x10, Multiloculated cyst on the wall of the urinary bladder lined with mucinous epithelium showing complex papillary architecture and luminal mucin (thick arrow). Unremarkable urothelium present (thin arrow) B&C. HE x100 and HE x200, Papillae lined with mucinous epithelium showing basally placed nuclei (thin arrow) and areas with low-grade dysplasia characterised by nuclear pseudo-stratification and hyperchromatic nuclei (thick arrow).

**Figure 3:**
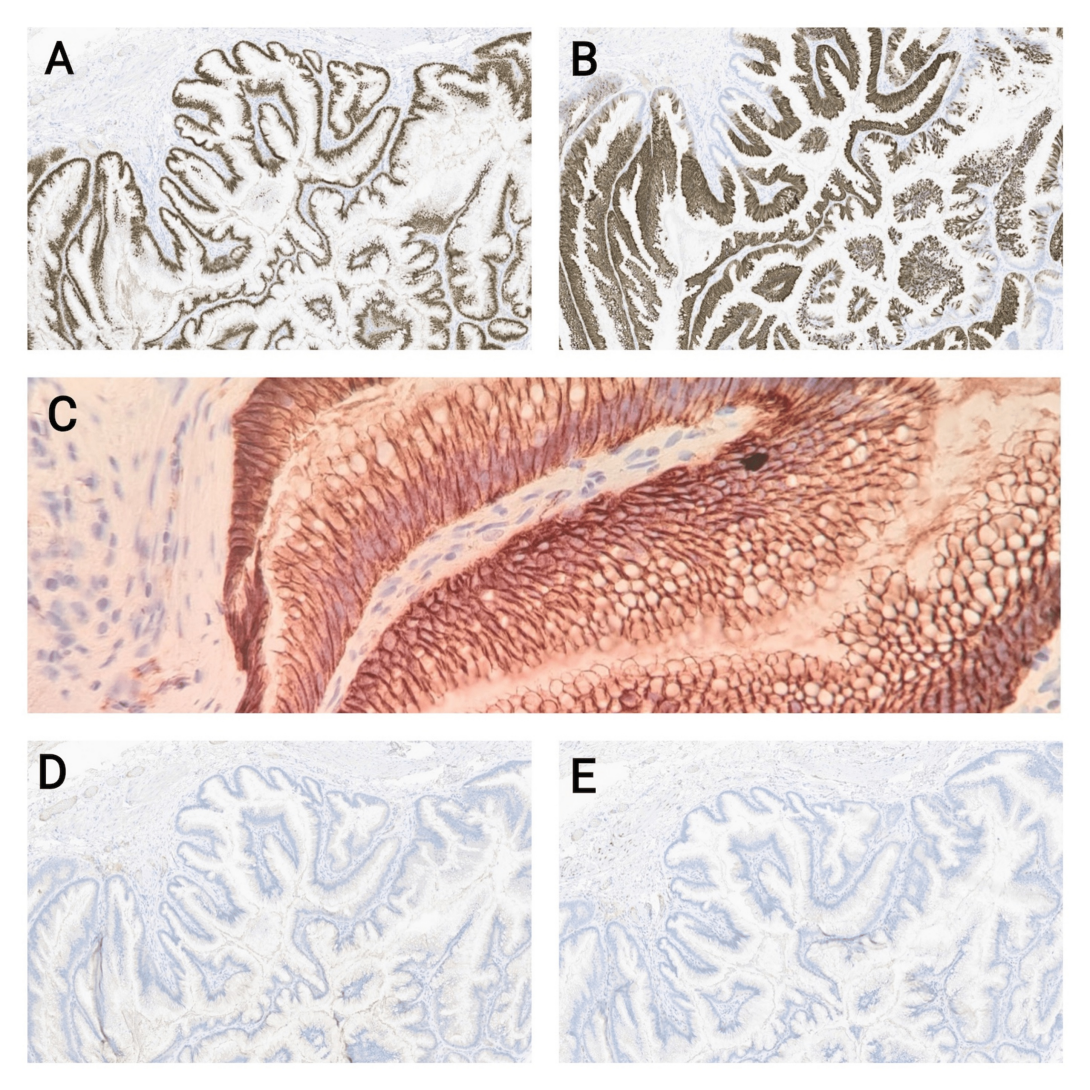
Immunohistochemistry. A. x100, the neoplastic cells are positive for CDX2 immunostain B. x100, they are positive for Cytokeratin 20 immunostain. C. x400, beta-catenin membranous expression with no nuclear localization. D, E. x100, the neoplastic cells are negative for Cytokeratin 7 and estrogen receptor.

Microscopic evaluation of haematoxylin and eosin-stained sections revealed a cystic lesion deep within the wall of the urinary bladder, surrounded by smooth muscle, and lined by columnar mucin-secreting epithelial cells predominantly arranged in a papillary pattern. Areas of low-grade dysplasia, characterised by epithelial proliferation, hyperchromatic nuclei, crowding and pseudo-stratification, were noted. The lumen contained mucin. No evidence of high-grade dysplasia, invasive malignancy, or extravasated mucin was observed. The urothelial lining of the bladder appeared within normal limits, and the lesion was completely excised with clear resection margins (Figures 2A-2C). Immunohistochemical staining demonstrated positivity of the mucinous epithelial lining for CK20 and CDX2, while being negative for CK7 and ER. Beta-catenin staining showed membranous expression with no nuclear localization (Figures 3A-3E). The morphology and immunoprofile were consistent with a urachal MCTLMP. Histopathological diagnosis was confirmed with a secondary read by St. James University Hospital, Leeds, UK.

## Discussion

This case, like most urachal remnants cases, was an incidental finding. Many authors report ultrasound to be substantial for diagnosis; however, much of this literature is based on paediatric populations [12]. However, CT is also thought to be suitable, and when there is concern of malignant transformation, it is the superior form of imaging. Despite combining different means of investigating the original cystic lesion, thorough histological examination is required to ascertain the final diagnosis and confirm the presence of a mucinous cystic tumour [13]. This emphasises the importance of surgical excision, with which we identify evidence of possible urachal-associated anomalies, for both completion of diagnostic workup and resolution to prevent sequelae of complications. Different surgical approaches have been described, including open, laparoscopic and robotic [14]. However, due to the rare incidence of urachal anomalies, there is minimal literature to substantiate that one method is superior to the other. Therefore, the decision on which approach to choose depends mainly on the surgeon’s experience or preference.

As far as histological findings are concerned, the specimen of our case grossly consisted of a cystic mass containing mucinous material with no solid areas. Microscopic examination of the entire specimen confirmed complete excision of the cystic lesion with clear surgical margins. The cyst was surrounded by smooth muscles and lined by columnar epithelium arranged in a papillary pattern with areas of low-grade dysplasia with no evidence of high-grade dysplasia or invasive malignant features which is consistent with the current WHO classification of mucinous cystic tumours of low malignant potential and further supports the diagnosis. With regard to immunohistological analysis, the specimen tested negative for CK7 and ER while testing positive for CK20 and CDX2, with Beta-catenin membranous expression with no nuclear localization. In general, mucinous cystic tumours of the urachus tend to all test positive for CK20, about 80% for CDX2 and only 30% are positive for CK7, while testing negative for nuclear β-catenin, oestrogen and progesterone receptors [9]. Therefore, the immunohistology staining in our case aligns with the findings in the current literature. Overall, the immunoprofile as well as the morphological features of the cysts in our case were consistent with intestinal-type differentiation with low malignant potential.

Given the potential risk of these tumours, surgical excision is warranted as it offers complete resolution with good outcomes [7]. Close initial follow-up is recommended due to the risk of malignant transformation [15]. But further treatment is typically not required after complete excision, unless recurrence or new symptoms arise.

## Conclusions

In a nutshell, MCTLPs are rare but potentially serious tumours of the urachus. Thorough clinical and radiological assessment as well as treatment by complete surgical excision with detailed histopathological analysis of these tumours is crucial to ensure the best outcome for the patients. Our case represents an asset to the scant literature on urachal tumours with low malignant potential and emphasises the importance of accurate diagnosis and management, preferably in an MDT setting. Moreover, we recommend further research in this area to hopefully be able to reach a standardised management approach for these rare tumours.
